# Development of a Secured IoT-Based Flood Monitoring and Forecasting System Using Genetic-Algorithm-Based Neuro-Fuzzy Network

**DOI:** 10.3390/s25133885

**Published:** 2025-06-22

**Authors:** Hero Rafael Castillo Arante, Edwin Sybingco, Maria Antonette Roque, Leonard Ambata, Alvin Chua, Alvin Neil Gutierrez

**Affiliations:** 1 Department of Electronics and Computer Engineering, De La Salle University, 2401 Taft Avenue, Malate, Manila 1004, Philippines; edwin.sybingco@dlsu.edu.ph (E.S.); antonette.roque@dlsu.edu.ph (M.A.R.); leonard.ambata@dlsu.edu.ph (L.A.); 2 Department of Mechanical Engineering, De La Salle University, 2401 Taft Avenue, Malate, Manila 1004, Philippines; alvin.chua@dlsu.edu.ph; 3 Department of Management and Organization, De La Salle University, 2401 Taft Avenue, Malate, Manila 1004, Philippines; alvin.gutierrez@dlsu.edu.ph

**Keywords:** LSTM, fuzzy inference system, genetic algorithm, flood prediction

## Abstract

The paper aims to provide a flood prediction system in the Philippines to increase flood awareness, which may help reduce property damage and save lives. Real-time flood status can significantly increase community awareness and preparedness. A flood model will simulate the flood level with secured data flow from the sensor to the cloud. The algorithms embedded in the flood predicting model include fuzzy logic, LSTM neural network, and genetic algorithm. The project used the Infineon security module (Infineon Technologies Philippines Inc., Metro Manila, Philippines) to create a secure connection from the setup to the AWS. All data transmitted were encrypted when being sent to AWS IoT Core, Timestream, and Grafana. After training and testing, the neuro-fuzzy LSTM network with genetic algorithm solution showed improved flood prediction accuracy of 92.91% compared to the ADAM solver that predicts every 2 h using an 0.02 initial learning rate, 1000 LSTM hidden layers, and 1000 epochs. The best solution predicts a flood every 3 h using an ADAM solver, a 0.01 initial learning rate, and 244 LSTM hidden layers for 158 epochs.

## 1. Introduction

Every year, the Philippines experiences heavy rains and severe weather conditions brought by typhoons or monsoons, which cause high levels of floods. Brown, muddy waters covered most communities and cities in the Philippines. Several towns are submerged. This natural disaster often leads to the loss of life, damages properties, and halts economic growth. In addition, climate change worldwide is making floods severe by having a longer duration of rainfall and increased precipitation. Aside from this, the water drainage systems, rivers, and dams have only a limited amount of water to hold. Therefore, if heavy rainfall occurs, it will contribute to more flooding.

Aside from the Philippines, several states in India also suffer from severe flooding every year. The problem in India is improper early warning signs and heavy storms during monsoons [1]. In Malaysia, natural disasters and human errors also cause floods. The poor design of drainage systems and heavy monsoon rains cause damage to property and loss of lives [2]. Malaysia and the Philippines have the same classification of floods: flash floods that occur without warning and monsoon floods caused by winds [3]. Nevertheless, floods will invariably cause damage and loss of lives if the community is unprepared or disseminates little information.

Several studies have made progress on flood monitoring and prediction systems. A study on smart flood monitoring systems used Internet of Things (IoT) and neural networks (NN) to enhance the scalability and reliability of the system. Moreover, the system correlates the measured parameters such as humidity, temperature, pressure, rainfall, and river water level [1]. In another study on flood monitoring systems using IoT with machine learning (ML), an ultrasonic sensor continuously monitors the water level and sends the data over the cloud using Arduino Uno and Raspberry Pi. People will receive alert messages on their phones whenever the water level reaches a critical level [2].

To enhance flood prediction, this system employs IoT for real-time data encryption and monitoring, forming the basis of the neuro-fuzzy network approach. Even though the Internet of Things cannot prevent floods, the system will provide real-time encryption of the information and warning signals that an incoming flood is about to happen based on gathered data. The system will use different sensors to measure atmospheric pressure, surrounding temperature, presence of rain, relative humidity, and flood level, providing information for the flood prediction system to forecast the upcoming flood. Also, information will be available in real time for public announcements and actions.

### 1.1. Review of Related Literature

In Maritime Southeast Asia, the Philippines is considered a medium vulnerability to flood disasters. Yet, along with the clusters of countries that belong to this echelon, the country has the lowest efficiency in managing floods, ranking fourth to Thailand, Myanmar, and Indonesia, respectively [4]. This being said, there has been an increase in journal articles published that optimize the use of IoT and camera sensors to improve flood management [5,6,7,8]. Despite this increase, the articles highlight the lack of research on floods affecting coastal areas where the Philippines, as an archipelago, contains cities in these areas. This was further corroborated by the work of Song [9], in their bibliometric literature review, sharing that articles focusing on flood monitoring have increased since 2017. They concluded from their review that flood monitoring should be managed using multiple data sources as well as providing real-time data through open-source software, websites, and free online services [10]. IoT has also helped store such data, a common practice in developed countries [7].

A systematic review and meta-analysis following the Preferred Reporting Items for Systematic Reviews and Meta-Analyses (PRISMA) protocol identified 60 studies investigating low-cost sensor technologies for monitoring coastal areas frequently affected by flooding. This body of work underscores the potential of affordable sensors in flood prediction and risk management [10,11].

In contrast, another line of research has focused on the utilization of sophisticated satellite imagery for flood monitoring, arguing that incorporating waterbody segmentation into image analysis can serve as an effective early-warning tool for flood-prone regions [12,13].

A further approach involves the deployment of drones for flood observation. However, floods in the Philippines are usually caused by typhoons, a meteorological occurrence that brings heavy rains and strong winds; thus, utilizing a drone to monitor them is quite a risk. No study has been made on whether drones can withstand high-speed winds and capture images while on flight [14].

In summary, the flood monitoring literature has gradually increased, proposing various methods for monitoring water levels and optimizing technology. This paper aims to contribute to the body of knowledge by incorporating various technologies to aid in the current situation of a developing country with many coastal areas that annually receive heavy rainfall and flooding. This research will not focus on the monitoring alone but rather on forecasting to enable potential victims to evacuate sooner to safer areas.

### 1.2. Current Practices

The research team met with the City Engineering Department of the Local Government Unit (LGU) on 28 May 2024 during their monthly meeting with various stakeholders. The meeting held in Century Park Hotel was attended by the Manila Electric Company, the Maynilad Water Services Inc., Consultants of the City Engineering Office—Woodlands Consultants, Inc., the education sector represented by various universities situated in the city, and Museo Pambata (a public museum for children), among others. The agenda of the meeting was the Drainage Masterplan of the City of Manila. The meeting addresses the prevalent issue of flooding in the city, which leads to regular work and school cancellations. Inviting various government agencies is significant because the drainage issue does not lie in the public works initiated by the LGU alone. The findings of the consultancy company present the issue of the other structures that will be impacted by the drainage management system owned by the water and electric companies. The consultancy company presented its progress reports, such as the selected priority areas for drainage in Manila, the various drainage management schemes, and its selection criteria. Table 1 presents the selection criteria.

Using Table 1 as the criterion for consideration holds significant implications for enhancing the effectiveness of drainage improvement schemes. The city’s drainage systems suffer from a lack of standardization—a direct result of contractors changing with every new LGU leadership over the years. In response to this challenge, consultants have proposed three distinct schemes that offer tailored solutions to address diverse local conditions while prioritizing both effectiveness and efficiency. These schemes are designed to implement area-specific improvements, with a scheme defined as a comprehensive plan or strategy aimed at enhancing the capacity and efficiency of a drainage system [4].

Building on the brief literature review and analysis of current practices, it becomes evident that there is a pressing need for a flood monitoring and forecasting framework that is both relevant and responsive to the realities of a developing country. Despite extensive research in the area, the question of whether flood monitoring can effectively predict or forecast floods remains unanswered in the literature. This paper seeks to address this gap by investigating flood prediction and monitoring using economical methods that can be readily adopted by the public sector in developing countries.

The remainder of this paper is organized as follows. First, we review related work on flood monitoring systems, flood prediction systems, and fuzzy logic. Next, the methodology used to integrate these systems is presented. Finally, the paper concludes with a discussion of the findings and an overview of relevant patents.

## 2. Related Works

### 2.1. Flood Monitoring Systems

Flood monitoring is a critical concern for many regions due to the potential for severe damage. Various studies have explored different approaches to designing effective flood monitoring systems such as wireless sensor networks, global positioning system (GPS), short message service (SMS), satellites, IoT, and artificial neural networks (ANN). The studies on wireless sensor networks allow the transmission of data and early warning signals over long distances that are essential for evacuation planning [15,16]. Different algorithms are used to assess the situations such as voting and decision tree [17]. Aside from this, some studies show the applications of GPS-based flood monitoring systems with alerts sent via SMS [18,19]. This approach ensures timely updates and alerts for residents, making it easier to respond to potential flooding situations.

The applications of the IoT have extended to flood monitoring systems because the Internet has become widely available to consumers. There are now several flood monitoring studies with IoT. Some studies monitor river levels that may affect nearby communities [1,2]. Additionally, other studies monitor road conditions affected by flood to provide alternative routes to decongest traffic [20].

Now, with the availability of Amazon Web Services (AWS), IoT-based flood monitoring data can be securely stored in the cloud. With data processing and analytics of flood data, rescue operations are possible with AWS [21]. In another study, Grafana in AWS was used to visualize data based on the water level inside the pipe [22]. Moreover, the formula for converting pressure inside the tube into height in meters is Equation (1).

(1) $$h_{T}=\frac{\left(P-P_{atm}\right)}{\rho g}+h_{1}-\frac{P_{atm}h_{1}}{P}+d_{3}$$

where $h_{T}$ is the total flood height, P is the measured air pressure by the DPS310 sensor, $P_{atm}$ is the atmospheric pressure, ρg is the specific gravity of water, $h_{1}$ is the height of the tube, and $d_{3}$ is the offset of the pipe from the ground.

Some studies tried monitoring flood levels with different parameters such as rainfall rate and soil moisture [23,24]. Lastly, it is essential to determine the differences, strengths, and weaknesses of flood monitoring and forecasting systems [25]. In a flood warning system, the dissemination speed of the warning is very important because of the small amount of time to implement emergency response. On the other hand, flood forecasting systems aim to provide reliable information and effective dissemination of information. The forecasting systems are usually designed on different algorithms and models.

### 2.2. Flood Prediction Systems

When ML is applied to flood monitoring systems, flood prediction becomes possible. There are different types and different architectures with varying depths created and designed for specific applications such as image recognition, speech recognition, approximation, regression, classification, time series forecasting, and natural language processing. Now, cameras have become a crucial tool in flood monitoring due to the variety of algorithms that can be applied for specific applications. These algorithms enhance the capabilities of cameras to detect, analyze, and interpret flood-related data effectively. For instance, a convolutional neural network (CNN) is used for image recognition and processing. It can analyze images to identify the status of rising water levels in gullies and drainages [26]. Additionally, it can classify 1-D data [27]. In some cases, CNN performs better than the support vector machine (SVM) and linear regression algorithms.

When sensors collect data over time, it is called time-series data. Predicting factors such as flood levels becomes accurate because of patterns in time. Thus, the timestamp of each datum is essential. Different studies on ML predict flood levels based on different parameters such as flood height readings of the ultrasonic sensor, flow rate, and weather patterns (temperature, humidity, atmospheric pressure, UV index, and rainfall intensity) [28,29,30]. When the Voting Classifier model is integrated with the random forest and extreme gradient boosting algorithm used to forecast flood at a rate of water, gauge height, turbidity, and temperature, the system is 99% accurate [31].

Other studies developed short-term flood prediction systems with artificial neural network-long short-term memory (ANN-LSTM) based on sensor readings [32]. The LSTM (long-short term memory) network is a specific type of recurrent neural network (RNN), which is a type of deep neural network (DNN). RNNs and LSTMs can remember the previous state and use it to predict the output, which is helpful for time series, text, and speech applications. Another system predicts 5 days into the future to control water management and irrigation [33]. Typically, root mean square error (RMSE) is used to check the performance of the neural network. Root mean square deviation (RMSD) was used to check the performance of the neural network [24]. It is possible to combine CNN and LSTM, called ConvLSTM, for flood forecasting [34].

Some papers compare the performance of different neural networks to determine the best algorithm. For instance, LSTM performed better than the AutoRegressive Integrated Moving Average (ARIMA) algorithm based on RMSE and mean absolute error (MAE) [35,36]. Another study showed that CNN-GRU (gated recurrent unit) performed better than ARIMA, WANN (wavelet-based ANN), and LSTM models [37]. Performance was based on the Nash–Sutcliffe efficiency coefficient (NSE), average relative error (MRE), and RMSE. Then, among the different multilayer perceptron (MLP) models (such as optimized MLP, MLP-GA (genetic algorithm), MLP-BA (bat algorithm), and MLP-BA-GA), the MLP-BA-GA performed the best [38]. Finally, the LSTM performed best when compared to MLP and GRU based on RMSE and NSE [36,39]. However, the hybrid system is better than LSTM [36]. This hybrid model uses the ADAM solver-based LSTM model followed by the ARIMA system. Another paper showed a hybrid system called a CNN-LSTM-ML hybrid prediction model for predicting groundwater levels in the Mojave Desert [40].

Among the papers discussed, a new type of loss function was introduced for the Timeseries Forecasting Model called DIstortion Loss including shApe and TimE (DILATE) [41]. Unlike the conventional performance metrics such as the RMSE loss function that predicts the average between the spike in data, this new loss function shows accurate predictions for sudden changes or spikes.

### 2.3. Reinforcement Learning

Machine learning (ML) is divided into three main categories, depending on the feedback signal or training data used in the system: supervised learning, unsupervised learning, and reinforcement learning [42]. First, supervised learning can predict the output by training the labeled datasets. Meanwhile, an unsupervised learning algorithm is when the datasets are neither labeled nor classified. This algorithm can provide output by drawing inferences and determining data patterns and groupings. Lastly, reinforcement learning is a category of ML that allows the system to determine the ideal behavior to maximize performance using simple reward feedback known as reinforcement signals. The outcome of the system came from current responses. In the study of urban flood modeling and flash flood management, IoT system reinforcement learning created a flood model based on the design parameters, output responses, and minimal losses [43,44].

### 2.4. Fuzzy Logic

Two widely used models in designing fuzzy logic are Mamdani and Takagi–Sugeno–Kang (TSK). In a paper about the framework of fuzzy rule interpolation, Zhang and Shen [45] described when to use Mamdani and TSK models. TSK models use polynomials as rule consequents where the results apply to solving regression problems. On the other hand, Mamdani models need defuzzification to obtain crisp results since antecedents and consequents represent fuzzy sets. There are different implementations of fuzzy logic combined with neural networks called “neuro-fuzzy systems” such as cooperative, concurrent, and adaptive [46]. In this study, the architecture for predicting flood uses the concurrent neuro-fuzzy system, wherein the inputs are preprocessed by fuzzy logic then the neural network processes the outputs.

An example of ANFIS (adaptive neural fuzzy inference system) architecture, a weather prediction system was developed with 100% accuracy [47]. The system predicts rain or no rain based on the input parameters such as temperature, humidity, and wind speed. Each input has three membership functions. Every membership function has three rules. Thus, there are 27 rules. Finally, each rule has its own corresponding output membership function. All 27 outputs are combined into one output that predicts the weather.

A fuzzy inference system can also assess an area’s flood hazard and risk based on flood area, flood depth, dependency ratio, and population density [48]. The parameters were fuzzified using a combination of triangular, trapezoidal, type-Z, type-S, and bell-shaped input membership functions to evaluate five distinct risk levels. Lastly, the accurate results of the flood risk analysis support effective decision-making.

### 2.5. Summary

Different studies have used sensors and IoT devices to develop flood monitoring systems. Based on the literature, flood monitoring systems are divided into several solutions: hardware-based systems, model-based systems, and hybrid systems. Hardware-based systems tend to tackle the problem by introducing sensors and microcontrollers that alert when a flood occurs. On the other hand, the researcher focuses on creating NN models or ML algorithms for predicting floods for model-based systems. Lastly, the solution mixes the alert and prediction systems for hybrid systems. The methodologies show the latest solutions and recommended approaches to the problem.

## 3. Methodology

**Figure 1:**
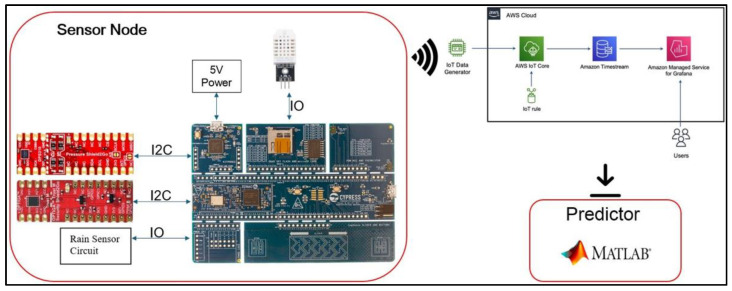
Model of the flood monitoring and prediction system.

The complete framework of the Flood Monitoring and Prediction System is shown in Figure 1. Flood prediction requires the sensor node to collect essential time-series data such as environment temperature, temperature inside the pipe, presence of rain, relative humidity, and flood level inside the pipe of the local area. The sensor node consists of a microcontroller, sensors, and a security module. The Cypress Semiconductor Programmable System-on-Chip (PSoC) Wi-Fi Board CY8CPROTO-062-4343W is the microcontroller of the sensor node (The microcontroller, DPS310 sensor, and the Optiga Trust M security module were sourced from Infineon Technologies Philippines Inc., Metro Manila, Philippines.) periodically monitors the sensors via the inter-integrated circuit (I2C) bus and general-purpose input/output (GPIO) ports and transmit the data to AWS—IoT Core, Timestream, and Grafana. Regarding flood height measurement, DPS310 senses the changes in atmospheric pressure inside the pipe of the simulated environment. Because of the Ideal Gas Law, the air pressure becomes proportional to the flood level when the flood closes the bottom of the pipe. When the flood level changes, the air inside the pipe is compressed by the water, which causes a change in air pressure. Also, the DPS310 sensor utilizes the I2C bus to communicate the data to the microcontroller.

On the other hand, some GPIO ports communicate with the following sensors: the DHT22 sensor (for humidity) and the rain sensor. Before sending data to the AWS, the microcontroller has to perform several procedures. Initially, the microcontroller should generate a certificate from the Optiga Trust M security module. The AWS verifies the certificate from the microcontroller. Then, the PSoC connects to the AWS via Wi-Fi. Every datum transmitted by the sensor node is protected from eavesdropping, tampering, and message forgery by the Optiga Trust M. Finally, the data is published on the AWS Managed Grafana and assessed by the flood prediction system. The sensor node is powered by a 5 V charger connected to a 220 V AC wall outlet to ensure reliable monitoring.

If there are problems with the Wi-Fi connection (no signal) or an unsuccessful data transfer, the microcontroller PSoC has an external reset pin. The microcontroller is programmed to trigger the reset pin via the GPIO pin. On the other hand, if a power outage occurs, all data are lost during the outage because no data can be transmitted to the AWS.

This paper simulated the monitoring of flood data. The simulated setup is placed in an open area where rain can be collected. In this experiment, the researcher created a flood level gauge using a container drum and DPS310 sensor. The container drum used is approximately 70 L, with two valves attached at the bottom for draining. The vertical pipe is 40 inches long. One end is open to let water in the pipe. The change in water level in the pipe is directly proportional to the atmospheric pressure read by the Infineon DPS310 (Infineon Technologies Philippines Inc., Metro Manila, Philippines).

### 3.1. Short-Term Flood Prediction System

**Figure 2:**
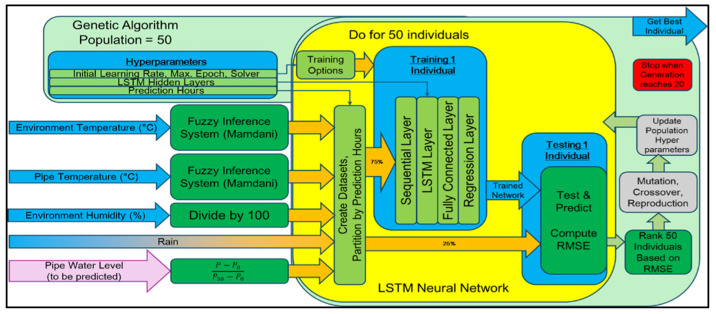
GA-based neuro-fuzzy network forecasting system.

Short-term flood prediction is generated hours before the impending flood. The algorithms used in making predictions are LSTM neural network, GA, and fuzzy logic. The latter two algorithms improve the accuracy of the results by optimizing the LSTM neural network hyperparameters based on the chosen performance metric and loss function and the normalization of all gathered data. Figure 2 illustrates the flow of creating a model for flood prediction. The different colors have no meaning.

Initially, all input data must be normalized. For the normalization of the temperatures, we used the architecture of a concurrent neuro-fuzzy system wherein the temperatures go into the fuzzy logic inference system, and the output is used as input for the LSTM neural network. With regards to the normalization of the humidity, since the humidity data is presented as a percentage, the data is simply divided by 100. Meanwhile, rain data has no changes because data is between 0 and 1. Lastly, the flood data is normalized by Equation (2) before proceeding to the LSTM neural network.

(2) $$P_{norm}=\frac{P-P_{0}}{P_{30}-P_{0}}$$

where $P_{norm}$ is the normalized flood height, P is the measured air pressure by the DPS310 sensor, $P_{0}$ is the atmospheric pressure (1013 hPa) at 0 inches flood height, and $P_{30}$ is the atmospheric pressure (1076.77 hPa) at 30 inches of flood height. Finally, Equation (3) presents the formula for calculating flood height in inches by multiplying Equation (1) by 39.3701.

(3) $$h_{T}=\left[\frac{\left(P-P_{atm}\right)}{\rho g}+h_{1}-\frac{P_{atm}h_{1}}{P}+d_{3}\;\right]*39.3701$$

where $h_{T}$ is the total flood height (inches), P is the measured air pressure by the DPS310 sensor in Pascals, $P_{atm}$ is the atmospheric pressure in Pascals, ρg is the specific gravity of water (9810 N/m^3^), $h_{1}$ is the height of the tube (0.84 m), and $d_{3}$ is the offset of the pipe from the ground (0.01 m).

**Figure 3:**
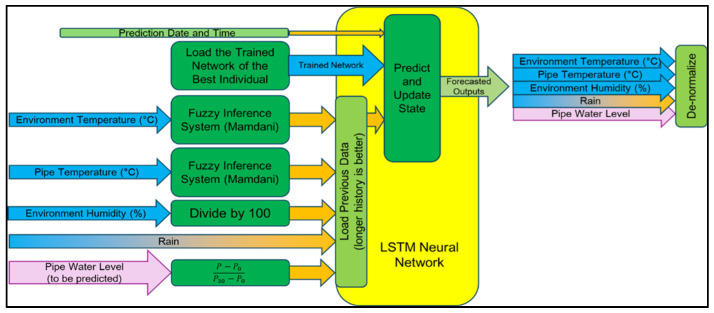
Optimized neural network for flood forecasting system.

During the neural network training, the accuracy of the prediction system increases because of the optimization of hyperparameters. One generation of a GA encompasses one LSTM neural network training. The GA will only stop optimizing the LSTM neural network when the generation has reached 20. After the GA is finished to determine the chromosomes of the best individual in the population, this set of hyperparameters can be used for the neural network forecasting system. Figure 3 illustrates the usage of the optimized neural network for flood forecasting. The different colors have no meaning. The trained network based on the hyperparameters of the best solution provided will create forecasts of the inputs based on the previously loaded data. Every time previous data is used to forecast, the trained network is updated to continuously make predictions.

#### 3.1.1. Fuzzy Inference System

**Figure 4:**
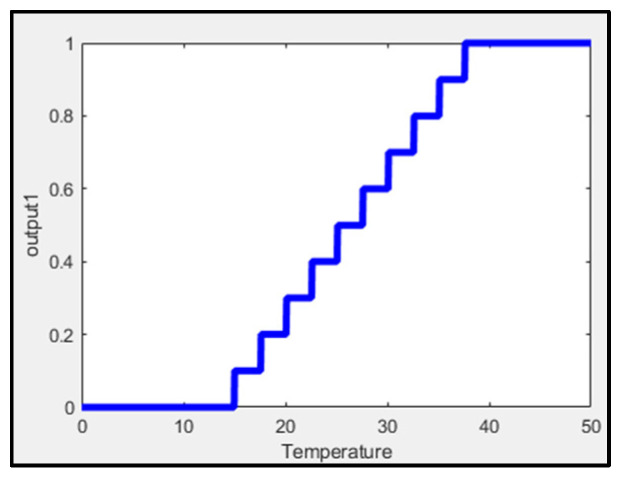
Mamdani FIS for temperature.

Fuzzy logic has been helpful in different applications, such as control systems, image processing, and robotics. This paper uses the Mamdani fuzzy inference system (FIS) to fuzzify environmental temperature and temperature inside the pipe. Normalizing the data improves learning speed and faster convergence. Figure 4 shows the fuzzification process of temperature, showing that each output level corresponds to a specific range of temperatures.

**Figure 5:**
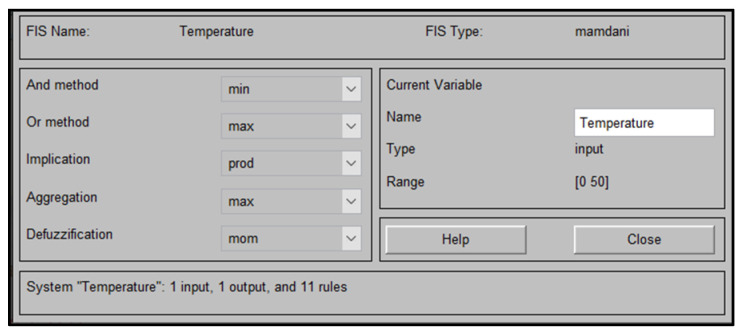
Temperature data preparation using Mamdani FIS.

Figure 5 shows the MATLAB R2020b FIS block diagram for applying temperature data to Mamdani FIS. Also, the figure includes the settings for fuzzification, aggregation, and defuzzification. During the fuzzification stage, the crisp input temperature value is converted to a fuzzy value by finding the degree of membership of the crisp input to the membership functions. Then, all membership-generated degrees are combined to form one summarized output in the aggregation stage. Finally, the aggregated result is defuzzified by using the defuzzication method mean of maximum (mom).

**Figure 6:**
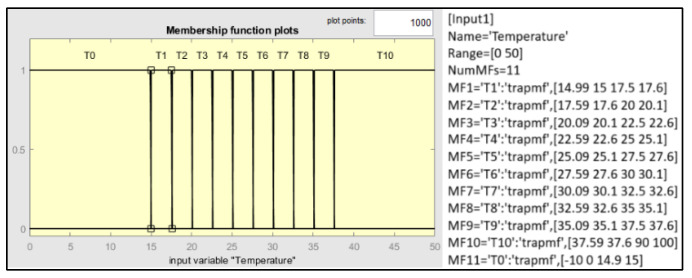
Configurations of the input membership functions.

The temperature FIS uses min, max, and moment of inertia for implication, aggregation, and defuzzification. The design of the temperature FIS uses eleven trapezoidal input membership functions, as shown in Figure 6.

**Figure 7:**
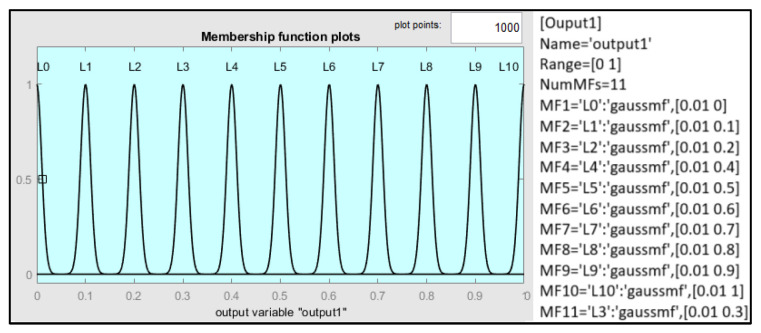
Configurations of the output membership functions.

Finally, the design uses eleven Gaussian output membership functions with a standard deviation of 0.01, as shown in Figure 7.

#### 3.1.2. Long Short-Term Memory (LSTM)

The LSTM algorithm is an RNN architecture used in deep learning, designed to process data in sequences. Moreover, LSTM networks recognize, detect, and predict time-based patterns. In this system, the LSTM algorithm predicts impending floods based on real-time data.

After all inputs have been normalized, we created two datasets for each partition dataset defined by prediction hours. The first dataset is [1 … n] length. The second dataset is [2 … n + 1] length. The first dataset will be used in the trained neural network model to create predicted outputs (Ypred). Conversely, the second dataset acts as the future values of the sensor node and contains future flood levels (Ytest). These are necessary for creating the neural network and measuring its performance.

**Figure 8:**
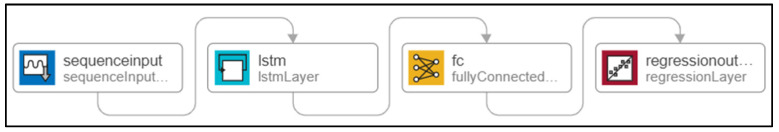
LSTM neural network architecture.

Figure 8 shows the base architecture for the LSTM neural network model. The first layer is the sequential layer because the inputs are time series data. The second layer is the LSTM layer, which is responsible for learning time series or sequential inputs. In this layer, the parameter for the number of hidden layers stores values that configure the model for flood prediction. The number of LSTM hidden layers is limited to 1000, as the system cannot compute the total parameters beyond this limit. The third layer is the fully connected layer, which maps the outputs of the last LSTM layer to five outputs. The last layer is the regression layer, which computes the RMSE to evaluate the performance of the system. After creating the regression model, the next step is to train the neural network.

During forecasting, the regression LSTM neural network evaluates its performance by calculating the difference between the predicted value and the actual value of the testing dataset. The root mean square error (RMSE) defines the performance metric in Equation (4). The RMSE measures the prediction errors or the difference of predicted output from the actual value. The GA finds the individual with the lowest (best) error.

(4) $$RMSE=\sqrt{\frac{\sum {\left(Y_{pred}-Y_{Test}\right)}^{2}}{N}}$$

where RMSE is the performance of the neural network model for a given testing dataset, $Y_{pred}$ is the predicted flood level of the trained LSTM neural network model from the first dataset, $Y_{Test}$ is the future flood level from the second dataset, and N is the length of the given testing dataset or prediction length.

#### 3.1.3. Genetic Algorithm (GA)

A genetic algorithm (GA) is a metaheuristic method inspired by natural selection to generate optimized solutions using biologically inspired operators such as mutation, crossover, and selection. This algorithm aims to produce a solution using natural selection, wherein the offspring’s inherited characteristics came from their parent’s characteristics in the previous generation. The GA will optimize the hyperparameters based on the computed RMSE between flood values in the testing dataset and predicted values. The following are the hyperparameters defined in this paper:

- Prediction minutes: random integer among {1, 5, 15, 30, 60, 90, 120, 180}.
- Type of solver: random number from 1 to 2. Stochastic gradient descent with momentum (SGDM) if value is {1}. Adaptive moment estimation (ADAM) if value is {2}.
- Initial learning rate: random number from 0.01 to 0.5.
- LSTM number of hidden layers: random number from 50 to 1000.
- Maximum number of epochs: random number from 50 to 200.

The first hyperparameter is the prediction minutes. With this hyperparameter, the system will predict the flood level for the next few minutes based on this value. The second hyperparameter indicates the solver to be used by the LSTM neural network.

The remaining three hyperparameters dictate the configuration of the LSTM neural network and its training options. The value of the fourth hyperparameter dictates the number of hidden layers of the LSTM layer. Finally, the third and fifth hyperparameters change the training options. The initial learning rate indicates the starting point of the learning rate. This will gradually decrease based on the learning rate factor and learning rate drop period. Lastly, the maximum number of epochs dictates the training duration.

In this paper, the GA will have 50 individuals working for 20 generations. Each individual has five chromosomes as an indication of hyperparameters. There will be no individuals with identical chromosomes for every generation in the current or previous generations. During each generation, each individual will be evaluated based on their fitness score (defined as a performance metric) and sorted from lowest fitness score (best) to highest fitness score (worst). The top 40 performing individuals earned the right to be retained in the population. The bottom ten are evicted from the population and replaced by the children of the top 10 individuals. The following are the mutation rules:

- For the top 10 individuals, no change in chromosome/hyperparameter.
- For top 11 to 40 individuals, three (3) random chromosomes mutate.
- For the ten new children, two (2) random chromosomes mutate.

## 4. Results and Discussion

### 4.1. The Datasets

**Figure 9:**
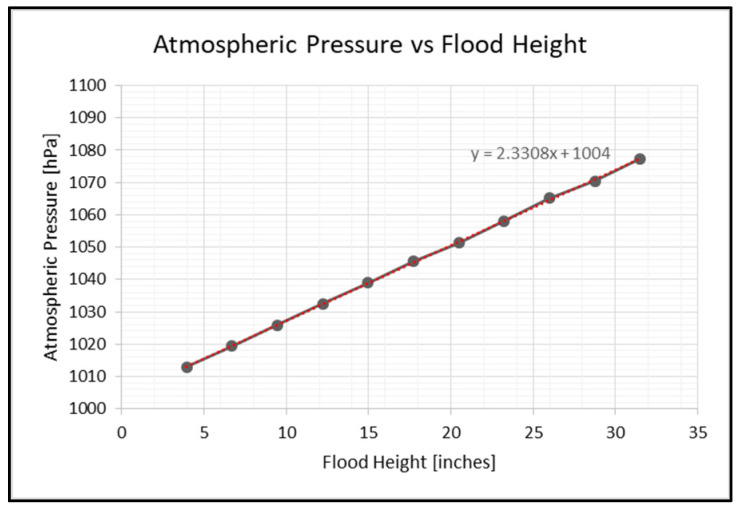
Relationship of pipe pressure to flood height.

This section discusses a simulated environment to test the flood prediction model. Figure 9 shows the relationship between atmospheric pressure and flood height. The equation y=2.3308x+1004 represents the best-fit line, where y corresponds to atmospheric pressure and x corresponds to flood height. The equation was obtained using the least squares regression method. The pipe is offset by 10 cm from the ground.

The datasets of atmospheric pressure, temperature, and presence of rain will be a time series. Designing the LSTM neural network model includes training, validating, and testing the gathered data from the cloud and sensor nodes. Different algorithms will help increase the performance of the NN model by tuning the parameters based on the datasets. Data was gathered on 23 March 2025 from 8:33 a.m. to 6:00 p.m. Philippine Standard Time. During this time, the sensor node transmits data every 3 s and up to two valves drain water from the drum. Each valve drains approximately 1.767 L per minute. With two valves open, the drum drains at a rate of approximately 3.534 L per minute.

**Figure 10:**
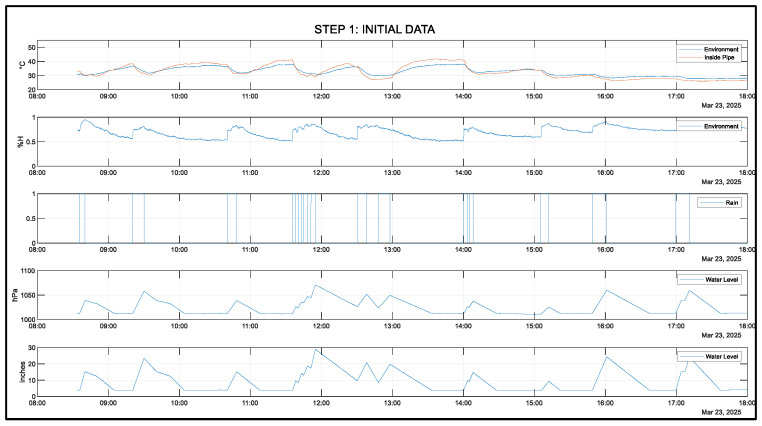
Complete data: 23 March 2025.

Figure 10 shows the gathered data. The first plot shows the temperature measurements inside the pipe and the sensor node environment. The second plot shows the humidity of the sensor node environment. The third plot shows the rain duration. The fourth plot shows the flood level inside the pipe. The fifth graph shows the flood level converted from atmospheric pressure into height. The water for this controlled environment is provided by a garden hose. Based on the gathered data, it can be observed that flood levels rise when humidity increases and it is raining, while the temperature decreases. When it stops raining, the humidity decreases while the temperature increases.

**Figure 11:**
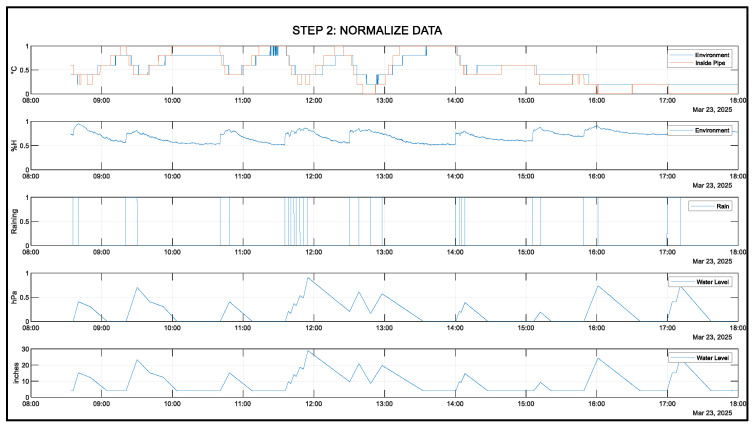
Normalized data: 23 March 2025.

Figure 11 shows the final preprocessed data of all gathered variables. All values are normalized and prepared for neural network training, testing, and validation.

### 4.2. Result: LSTM Neural Network (Without Genetic Algorithm)

**Figure 12:**
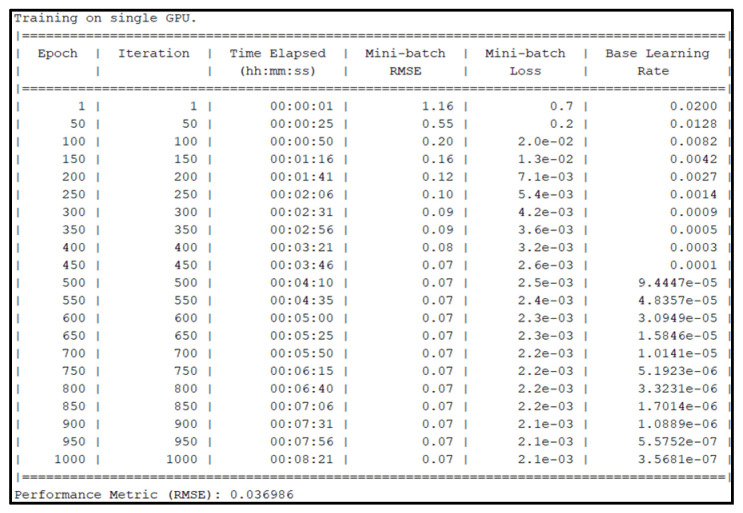
Training results of 1000 epochs (regression).

In this section, the prepared data are partitioned every 120 min to form datasets. These datasets are used to predict the next 120 min using the LSTM neural network model that has 1000 LSTM hidden layers and ADAM solver with a 0.02 initial learning rate for 1000 epochs. The training, testing, and verification were executed on the GPU NVIDIA GeForce RTX2070 with Max-Q Design, and the training results are illustrated in Figure 12.

Based on the results, the actual training time was 8 min and 21 s. Aside from the time parameters, performance metrics of mini-batch RMSE and mini-batch loss are displayed. The objective was to create a regression neural network with an ideal training RMSE of 0. After the training of the regression model, the performance of the neural network was 0.036986.

**Figure 13:**
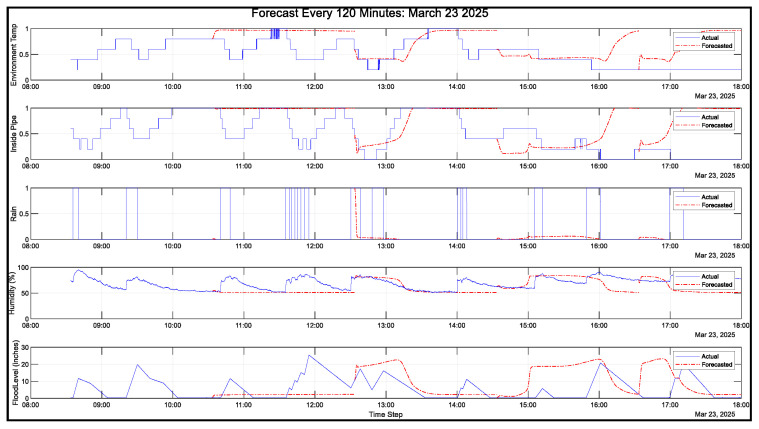
Closed loop forecasting: 23 March 2025.

Figure 13 shows the closed loop forecasting results on 23 March 2025 from 8:33 a.m. to 6:00 p.m. for the 2 h prediction. The blue line indicates the actual data gathered by the sensor node. On the other hand, the red dashed line indicates the flood predicted by the best solution.

### 4.3. Result: LSTM Neural Network (Genetic Algorithm)

**Figure 14:**
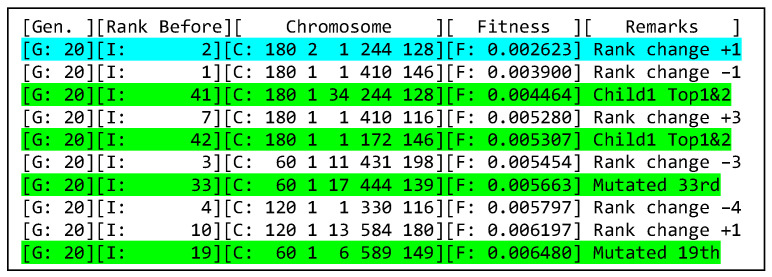
Top 10 of Generation 20.

In this section, the GA aimed to find the best combination of the hyperparameters based on the lowest performance metric. The performance metric was the calculated RMSE between the flood level data of the testing dataset and the predicted values generated by the newly trained network. After 6 h and 57 min of running the program in MATLAB R2020b, the top 10 individuals and their fitness scores from generation 1 to 20 are displayed in Figure 14. Highlighted in Green are the new ones in the top 10. The best solution is highlighted in Turquoise.

**Figure 15:**
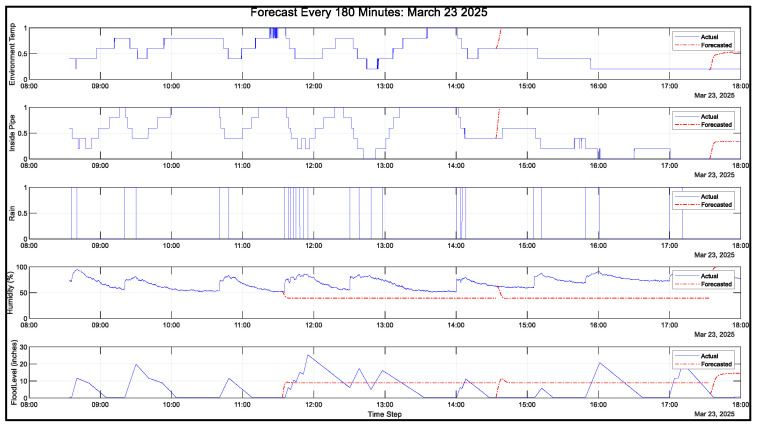
Closed loop forecasting (23 March 2025).

Based on the results of 20 generations, the GA suggests predicting flood level every 180 min using an ADAM solver with 244 LSTM hidden layers trained with a 0.01 initial learning rate for 128 epochs. Figure 15 shows forecasted results on 23 March 2025 for the best solution. The blue line indicates the actual data gathered by the sensor node. On the other hand, the red dashed line indicates the flood predicted by the best solution.

### 4.4. Data Analysis

**Figure 16:**
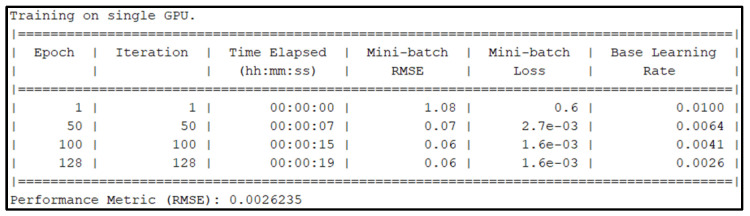
Training results of the best solution.

This section discusses the results of the statistical methods employed to evaluate the performance of the developed flood forecasting system. The first analysis focuses on the system’s accuracy. The first LSTM neural network, without GA optimization, used 1000 hidden layers with an ADAM solver. It predicts every 120 min and was trained with an initial learning rate of 0.02 for 1000 epochs. The training time was 8 min and 21 s, which yielded a performance metric of 0.036986. In contrast, the second LSTM neural network, optimized with a GA, utilized 244 hidden layers with an ADAM solver. It predicts every 180 min, trained with an initial learning rate of 0.01 for 128 epochs. When trained in isolation, this configuration required only 19 s of training time and achieved a performance metric of 0.0026235, as shown in Figure 16. Table 2 provides a detailed comparison of the two LSTM neural networks. The solution provided by the GA has an accuracy improvement of 92.91%.

Subsequently, a T-test analysis was conducted to identify significant differences in the performance of the two systems. This analysis utilized MATLAB’s T-Test function (ttest2), allowing for the calculation of the *p*-value, test statistic, confidence interval, and hypothesis test results. Upon checking and comparing the RMSE values or the accuracies of the two LSTM neural network systems, there was a significant difference between the two LSTM neural networks. T-test results and analysis are provided in Table 3.

Based on the analysis and results from accuracy and T-test, it can be concluded that there was a statistically significant difference in the performance of the two LSTM neural networks. The variations in their configurations in the number of hidden layers, solvers, and optimization techniques show that the prediction capabilities provided by the GA-optimized LSTM offer computational advantages with reduced training time and fewer hidden layers and provide better practical solutions for computational efficiency, reduced power consumption, and strong accuracy.

## 5. Conclusions

The created secured IoT-based flood monitoring and forecasting system using a GA based neuro-fuzzy network addresses the Philippines’ lack of flood prediction systems. The sensor node gathers atmospheric pressure, temperature, rain, and flood level data. Moreover, the Infineon CY8CPROTO-062-4343W PSoC™ 6 Wi-Fi BT Prototyping Kit (Infineon Technologies Philippines Inc., Metro Manila, Philippines) handles data and network communications. Regularly, data is transmitted in real time on Amazon Web Services. A secured data connection has been established between the sensor node and the AWS using the certificates and keys. Moreover, only the registered sensor nodes with the registered certificate from Infineon Optiga Trust M (Infineon Technologies Philippines Inc., Metro Manila, Philippines) can communicate with AWS via the MQTT protocol.

For flood prediction, this study employed a regression LSTM neuro-fuzzy model. Before inputting data into the LSTM neural network, all variables were normalized, with temperature data specifically normalized using fuzzy logic inference systems. The model predicts imminent flooding based on its training, and the GA was used to optimize hyperparameters for the prediction model.

With regards to flood prediction results, the forecasting system evaluated on both a 1-month dataset and a simulated controlled environment dataset demonstrated superior computational efficiency, reduced power consumption, and higher accuracy when optimized by the GA. Across all 20 generations, the ADAM solver emerged as the most effective for flood prediction. The final system successfully forecasts flooding 2 to 3 h in advance. It is recommended that for some rapid-onset or short-duration extreme flood events, a lead time of 2 to 3 h may be insufficient.

## Figures and Tables

**Table 1:** Technical factors for Manila City drainage assessment (MCDA) (Woodlands Consultants, Inc. 24 May 2024) [4].

Technical Factors for Selection Criteria	Description
Technical complexity	deals with the complexity of design, construction methods, and the technology involved
Adaptability	deals with the capacity for long-term adjustments, including accommodating future expansions, changes in the use, and integrating emerging technologies
Flexibility	deals with the immediate responsiveness to short-term changes, allowing for adjustments in flow rates, component operations, and operational strategies
Lifetime	deals with the durability and longevity in terms of the quality of materials used, construction standards, and maintenance practices to determine the expected lifespan of the infrastructure
Maintenance requirements	deals with the long-term maintenance requirements, including the frequency and complexity of the maintenance activities needed to ensure the continued functionality and performance of the drainage system.

**Table 2:** Comparison summary of the two LSTM neural networks.

Hyperparameters	LSTM Without GA	LSTM with GA
Type of solver	ADAM	ADAM
LSTM hidden layers	1000	244
Epoch	1000	128
Prediction minutes	120	180
Initial learning rate	0.02	0.01
Training time	8 min 21 s	19 s
RMSE	0.036986	0.0026235

**Table 3:** T-Test Result and Analysis.

Statistical Parameters	Criteria for Significant Difference	Result
*p*-Value	<0.05	0
Confidence interval	Does not include 0	[2.8664, 2.8664]
Hypothesis test result	1	1

## Data Availability

The original data presented in the study are openly available in https://github.com/Dragonex68/-sensors-25-03885, accessed on 1 May 2025.
